# IMPACT OF DEPRESSIVE SYMPTOMS ON ORAL HEALTH OF OLDER ADULTS WITH CHRONIC CONDITIONS BY GENDER

**DOI:** 10.1093/geroni/igad104.3411

**Published:** 2023-12-21

**Authors:** Dahye Hong, Jennifer (Jeehyun) Kim, Bada Kang

**Affiliations:** Yonsei University, Seoul, Republic of Korea; Yonsei University, Seoul, Republic of Korea; Yonsei University, Seoul, Republic of Korea

## Abstract

Oral health is crucial for the nutrition and well-being of older adults, especially those with chronic conditions. Although recent studies have demonstrated an association between depressive symptoms and oral health, little is known regarding the gender-specific association between symptoms of depression and oral health among older Korean adults with chronic conditions. Therefore, this study examined the gender-specific relationship between depressive symptoms and oral health, using data from the Korean Longitudinal Study of Aging (2018–2020). Study participants aged 65 or older with one or more chronic conditions were analyzed (N = 1,185 women and 1,919 men). The comprehensive aspects of oral health, including functional limitations, pain, discomfort, and psychological and behavioral impacts, were measured. Generalized Estimation Equation analyses were applied to two models presenting depressive symptoms dichotomously and the severity of depression, respectively. When considering depressive symptoms in dichotomous manner, such as present or absent, having three or more chronic conditions and experiencing cognitive decline were both significantly related to deteriorated oral health for both genders. Moreover, the association between depressive symptoms and deteriorated oral health was more robust in men. While categorizing the severity of depressive symptoms, the worse the depressive symptoms, the poorer the oral health in women. Therefore, this study suggests that interventions to alleviate depressive symptoms could benefit the oral health of both men and women with multiple chronic conditions. For women, targeted interventions based on the severity of depressive symptoms could effectively improve oral health.

